# Acyl-CoA-binding and self-associating properties of a recombinant 13.3 kDa N-terminal fragment of diacylglycerol acyltransferase-1 from oilseed rape

**DOI:** 10.1186/1471-2091-7-24

**Published:** 2006-12-27

**Authors:** Randall J Weselake, Milan Madhavji, Steve J Szarka, Nii A Patterson, William B Wiehler, Cory L Nykiforuk, Tracy L Burton, Parveen S Boora, Steven C Mosimann, Nora A Foroud, Benjamin J Thibault, Maurice M Moloney, André Laroche, Tara L Furukawa-Stoffer

**Affiliations:** 1 Department of Agricultural, Food and Nutritional Science, 4-10 Agriculture/Forestry Centre, University of Alberta, Edmonton, Alberta, T6G 2P5, Canada; 2 Department of Chemistry and Biochemistry, University of Lethbridge, Lethbridge, Alberta, T1K 3M4, Canada; 3 Department of Biological Sciences, University of Calgary, Calgary, Alberta, T2N 1N4, Canada; 4 Lethbridge Research Centre, Agriculture and Agri-Food Canada, Lethbridge, Alberta P.O. Box 3000 Main, Lethbridge, Alberta, T1J 4B1, Canada; 5 Present address : SemBioSys Genetics Inc., 110, 2985 23 Avenue N.E., Calgary, AB T1Y 7L3, Canada; 6 Present address: Metabolix Inc., 21 Erie Street, Cambridge, MA 02139, USA

## Abstract

**Background:**

Diacylglycerol acyltransferase (DGAT, EC 2.3.1.20) catalyzes the acyl-CoA-dependent acylation of *sn*-1, 2-diacylglycerol to generate triacylglycerol and CoA. The deduced amino acid sequence of cDNAs encoding DGAT1 from plants and mammals exhibit a hydrophilic N-terminal region followed by a number of potential membrane-spanning segments, which is consistent with the membrane-bound nature of this enzyme family. In order to gain insight into the structure/function properties of DGAT1 from *Brassica napus *(BnDGAT1), we produced and partially characterized a recombinant polyHis-tagged N-terminal fragment of the enzyme, BnDGAT1_(1–116)_His_6_, with calculated molecular mass of 13,278 Da.

**Results:**

BnDGAT1_(1–116)_His_6 _was highly purified from bacterial lysate and plate-like monoclinic crystals were grown using this preparation. Lipidex-1000 binding assays and gel electrophoresis indicated that BnDGAT1_(1–116)_His_6 _interacts with long chain acyl-CoA. The enzyme fragment displayed enhanced affinity for erucoyl (22:1cis^Δ13^)-CoA over oleoyl (18:1cis^Δ9^)-CoA, and the binding process displayed positive cooperativity. Gel filtration chromatography and cross-linking studies indicated that BnDGAT1_(1–116)_His_6 _self-associated to form a tetramer. Polyclonal antibodies raised against a peptide of 15 amino acid residues representing a segment of BnDGAT1_(1–116)_His_6 _failed to react with protein in microsomal vesicles following treatment with proteinase K, suggesting that the N-terminal fragment of BnDGAT1 was localized to the cytosolic side of the ER.

**Conclusion:**

Collectively, these results suggest that BnDGAT1 may be allosterically modulated by acyl-CoA through the N-terminal region and that the enzyme self-associates via interactions on the cytosolic side of the ER.

## Background

In eukaryotes, triacylglycerol (TAG) is synthesized through both acyl-CoA-dependent and acyl-CoA-independent processes [1]. Acyl-CoA:diacylglycerol acyltransferase (DGAT, EC 2.3.1.20) catalyzes the acylation of *sn*-1,2-diacylglycerol (DAG) to generate TAG and CoA. Various acyl donors have also been identified in the acyl-CoA independent acylation of DAG including a second DAG molecule [2,3], phosphatidylcholine [4,5]and free fatty acid [6]. Studies on the acyl-CoA-dependent acylation of TAG, however, have been more extensive since this pathway has been recognized for several years. Two families of DGAT, designated DGAT1 and DGAT2, have been identified with homologues in animals, plants and various microorganisms [7]. DGAT1 cDNA was first cloned from *Mus musculus *liver [8] and shortly thereafter from developing seeds of *Arabidopsis thaliana *[9-12] and microspore-derived cell suspension cultures of *Brassica napus *[13-15]. Recently, DGAT1 cDNA has been cloned from other plant sources including developing castor bean (*Ricinus communis*) [16] and seeds of burning bush (*Euonymus alatus*) [17]. Deduced amino acid sequences of members of the DGAT2 gene family, however, have been shown to share no homology with the DGAT1 family [7]. It is important to note that a second cDNA encoding a *B. napus *DGAT, which represented a truncated form of DGAT1, has been previously designated DGAT2 [13-15]. The hydropathy plots of DGAT1 from various species are very similar in that the polypeptides all display a hydrophilic N-terminal segment followed by a number of potential transmembrane intrapolypeptide segments [9,13,16,18]. The general characteristic of the DGAT1 hydropathy plot is also very similar to that of type 1 acyl-CoA:cholesterol acyltransferase (ACAT, EC 2.3.1.26), which uses cholesterol as an acyl acceptor instead of DAG [18]. Structure/function relationships in ACATs have been more fully elucidated than for DGATs. For example, the topologies of ACAT1 and ACAT2 have been determined and have provided valuable insights into the operation of this enzyme [19,20].

The current study was undertaken to gain insight into the structure and function properties of *B. napus *DGAT1 (BnDGAT1) using a poly-His tagged recombinant hydrophilic N-terminal region of the enzyme which is designated BnDGAT1_(1–116)_His_6_. The recombinant enzyme fragment interacted with acyl-CoA displaying positive cooperativity and self-associated to form a dimer and tetramer, which was consistent with recent reports on the self-association of human DGAT1 [21] and mammalian ACAT1 [22].

## Results

### Expression, purification and crystallization of BnDGAT1_(1–116)_His_6_

**Figure 1:**
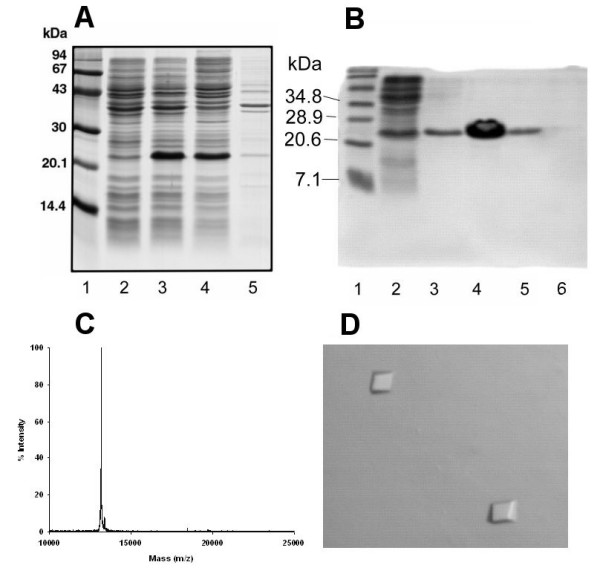
Expression, purification and crystallization of BnDGAT1_(1–116)_His_6_. Panel A: Induction of BnDGAT1_(1–116)_His_6 _expression in bacteria monitored by SDS-PAGE. From left to right: molecular mass markers (lane 1), clarified lysate from non-induced bacteria (lane 2), clarified lysate from two independent incubations of bacteria treated with IPTG (lanes 3 and 4), pellet obtained from clarified lysate of bacteria induced with IPTG (lane 5). Experiments to demonstrate induction of BnDGAT1_(1–116)_His_6 _synthesis were conducted using an incubation medium of 5 ml (instead of the 500 mL used for preparative work). Harvested *E. coli *were resuspended in 100 mM phosphate buffer (pH 7.4) containing 150 mM NaCl. The suspension was passed through a French pressure cell at 20,000 p.s.i. three times and the ruptured bacteria were centrifuged at 13,000 *g *for 15 min to obtain a clarified lysate. The 13,000 *g *pellet obtained from ruptured bacteria that had been induced with IPTG was resuspended in extraction buffer. Equal volumes of each sample were applied to the SDS gel. Panel B: Analysis by SDS-PAGE of BnDGAT1_(1–116)_His_6 _purified by immobilized nickel ion chromatography. From left to right: molecular mass markers (lane 1), pass-through fraction (lane 2), fraction eluted with equilibration buffer (lane 3), and fractions eluted with 4 ml portions of equilibration buffer containing 40, 60 and 80 mM imidazole, respectively (lanes 4–6). The protein load for lane 4 was about 10 μg. Ten microliter aliquots of each of the eluents were applied to the gel. Panel C: MALDI-TOF spectrum of BnDGAT1_(1–116)_His_6_. The spectrum was acquired using a Voyager DE-Pro TOF MS (Applied Biosystems, Foster, CA, USA) using a 2-layer spotting technique. Matrix layer 1 was approximately 15 mg/mL α-cyano-4-hydroxycinnamic acid dissolved in 1:2 (v/v) methanol:acetone. Matrix layer 2 was saturated α-cyano-4-hydroxycinnamic acid in 1:2 (v/v) methanol/water. One microliter of layer 1 solution was spotted and allowed to dry. Protein sample (450 μg/mL) was mixed with layer 2 solution in a ratio between 1:1 and 1:4 (sample:matrix). One microliter of layer 2 solution was spotted on the dried layer 1 spot, and allowed to dry. The dried spot was washed 2–3 times with a drop of cold distilled water to remove salts prior to analysis by MALDI-TOF mass spectrometry. Panel D: Microcrystals of BnDGAT1_(1–116)_His_6_. To begin preparation of microcrystals, a 12 mg/mL solution of BnDGAT1_(1–116)_His_6 _was dialyzed against 10 mM glycine-NaOH (pH 9.0) containing 20 mM NaCl and 1 mM EDTA. Initial screening for crystallization conditions using vapor diffusion and hanging drops resulted in several instances of microcrystals, which grew as plates, within 1–2 weeks. Microcrystals were obtained from ammonium sulfate and ammonium phosphate solutions over a wide pH range.

A very brief description of the procedure for generating BnDGAT1_(1–116)_His_6 _was presented previously in a conference proceeding document [23]. A hydropathy plot [24] of the deduced amino acid sequence of BnDGAT1 [13] indicated that the N-terminal region of the enzyme was relatively hydrophilic compared to the rest of the protein. Based on this information, we designed a construct which would result in the production of a gene product consisting of the first 116 amino acid residues of BnDGAT1 followed by a four amino acid residue (AALE) linker and 6 His residues. Automated DNA sequencing was performed across the cloning junctions and for the entire coding region, to verify there was no shift in the open reading frame. Induction of BnDGAT1_(1–116)_His_6 _synthesis by isopropyl β-D-thiogalactoside (IPTG) treatment of bacteria is shown in Figure 1A. Analysis of lysates from induced *Escherichia coli *(lanes 3 and 4) clearly showed the appearance of an abundant polypeptide with an apparent molecular mass of about 23 kDa, which was considerably larger than the calculated molecular mass of 13,278 Da for BnDGAT1_(1–116)_His_6_. The 23 kDa band was barely perceptible in the pellet protein (lane 5), indicating that the expressed gene product was soluble in the extraction buffer. The poly-His tag on BnDGAT1_(1–116)_His_6 _facilitated effective purification of the recombinant N-terminal fragment by immobilized nickel ion chromatography. Analysis of different elution fractions of the Ni^2+^-NTA-agarose column by SDS-PAGE is depicted in Figure 1B. The protein fraction that was eluted with 40 mM imidazole was highly purified based on SDS-PAGE (lane 4) and thus was used for subsequent characterization work. Analysis of purified BnDGAT1_(1–116)_His_6 _by MALDI-TOF mass spectrometry indicated a molecular mass of about 13.2 kDa, which was in close agreement with the calculated molecular mass (Figure 1C). Analysis of BnDGAT1_(1–116)_His_6 _by dynamic light scattering (DLS) indicated that the protein preparation was largely monodisperse and potentially suitable for crystallization. Small plate-like monoclinic crystals were grown from preparations of purified BnDGAT1_(1–116)_His_6 _(Figure 1D). The largest microcrystals, however, were too small (0.07 × 0.07 × 0.01 mm) for x-ray diffraction studies using a rotating anode x-ray source.

### BnDGAT1_(1–116)_His_6 _interacts with long chain acyl-CoAs in a cooperative fashion

**Figure 2:**
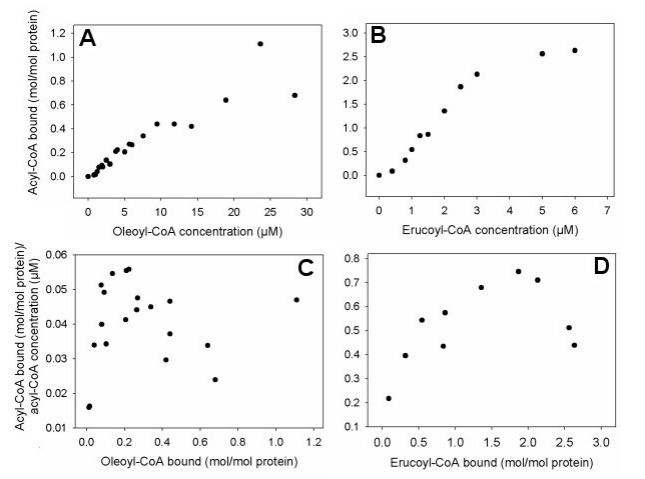
Binding of acyl-CoAs to BnDGAT1_(1–116)_His_6_. Panels A and B: Binding of oleoyl-CoA or erucoyl-CoA to BnDGAT1_(1–116)_His_6_. Lipidex-1000 binding assays were conducted in duplicate in 10 mM potassium phosphate buffer, pH 7.4, at 30°C with [1-^14^C]acyl-CoAs and 0.2 μM protein. Incubations were set up without protein to correct for acyl-CoA that was not adsorbed by the Lipidex-1000. Data points represent mean values. Panels C and D: Scatchard plot [26] analysis of the binding of 18:1-CoA or 22:1-CoA to BnDGAT1_(1–116)_His_6_.

In a previous study, we demonstrated that [1-^14^C]oleoyl-CoA interacted with BnDGAT1_(1–116)_His_6 _using a low concentration range (0–5 μM) of thioester [23]. In the current study, the range of thioester concentrations used in Lipidex-1000 binding assays was extended up to near 30 μM for [1-^14^C]oleoyl-CoA (Figure 2A). As well, binding studies were conducted with [1-^14^C]erucoyl-CoA for comparative purposes. Binding experiments with BSA, which is known to interact with acyl-CoAs [25], demonstrated that the Lipidex-1000 assay was effective and thus served as a positive control (data not shown). In contrast, we have previously shown that ovalbumin does not bind acyl-CoA using this assay [23]. The binding of both acyl-CoA species to BnDGAT1_(1–116)_His_6 _occurred in a sigmoidal fashion. The binding ratio of thioester: BnDGAT1_(1–116)_His_6 _was based on the molecular mass of 13,278 Da calculated from the amino acid sequence of the N-terminal fragment. BnDGAT1_(1–116)_His_6 _bound more erucoyl-CoA than oleoyl-CoA at considerably lower concentrations of acyl-CoA indicating that the N-terminal fragment had a greater affinity for erucoyl-CoA than oleoyl-CoA. Analysis of the binding data using Scatchard plots [26] is depicted in Figures 2C and 2D. Both plots rose to a maximum and then decreased suggesting that the interaction between acyl-CoA and BnDGAT1_(1–116)_His_6 _involved positive cooperativity. Dissociation constants of about 17 μM and 2 μM were determined for the binding of oleoyl-CoA and erucoyl-CoA, respectively, to the high affinity state of BnDGAT1_(1–116)_His_6_. The dissociation constants were determined using the data points constituting the negative slopes of the Scatchard plots.

**Figure 3:**
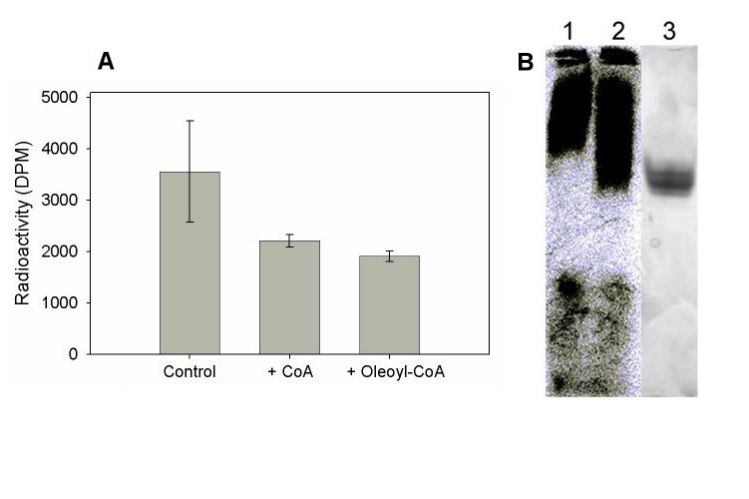
Effect of CoA on binding of acyl-CoA to BnDGAT1_(1–116)_His_6 _and demonstration of acyl-CoA binding using electrophoresis. Panel A: Effect of non-radiolabeled CoA (8 μM) or oleoyl-CoA (8 μM) on the binding of [1-^14^C]oleoyl-CoA to BnDGAT1_(1–116)_His_6_. Each incubation mixture contained 0.2 μM protein and 19 μM radiolabeled acyl-CoA. Following sedimentation of Lipidex-1000, equal aliquots of the supernatants were assayed for radioactivity. Each incubation was performed in triplicate and each data point represents the mean ± SD. Panel B: Binding of acyl-CoA to BnDGAT1_(1–116)_His_6 _demonstrated using non-denaturing gel electrophoresis and phosphorimaging. Phosphorimage obtained using radiolabeled erucoyl-CoA (lane 1) and BnDGAT1_(1–116)_His_6 _incubated with radiolabeled erucoyl-CoA (lane 2). Protein stain of BnDGAT1_(1–116)_His_6 _incubated with radiolabeled erucoyl-CoA is shown in lane 3.

A Lipidex-1000 competition binding study was conducted by introducing unlabeled compounds into a binding medium containing 19 μM [1-^14^C]oleoyl-CoA. Test compounds included 8 μM free CoA and 8 μM oleoyl-CoA. A previous study indicated that micromolar concentrations of CoA were effective in stimulating microsomal DGAT activity from microspore-derived cell suspension cultures of oilseed rape [27], so it seemed logical to test the possible effect of these compounds on the binding of thioester. The presence of either free CoA or oleoyl-CoA was effective in displacing a similar quantity of radiolabeled ligand from BnDGAT1_(1–116)_His_6 _(Figure 3A).

Additional evidence for the interaction of BnDGAT1_(1–116)_His_6 _with acyl-CoA was obtained using non-denaturing gel electrophoresis. When the fragment was preincubated with an equimolar concentration of radiolabeled erucoyl-CoA followed by non-denaturing gel electrophoresis and phosphorimaging, radiolabel was extended to the position of BnDGAT1_(1–116)_His_6 _in the gel (Figure 3B). The rather broad band seen in the protein stain (lane 3) suggested the presence of at least two closely associated polypeptides, which probably represent different association states of BnDGAT1_(1–116)_His_6 _(see next section).

### BnDGAT1_(1–116)_His_6 _self-associates

**Figure 4:**
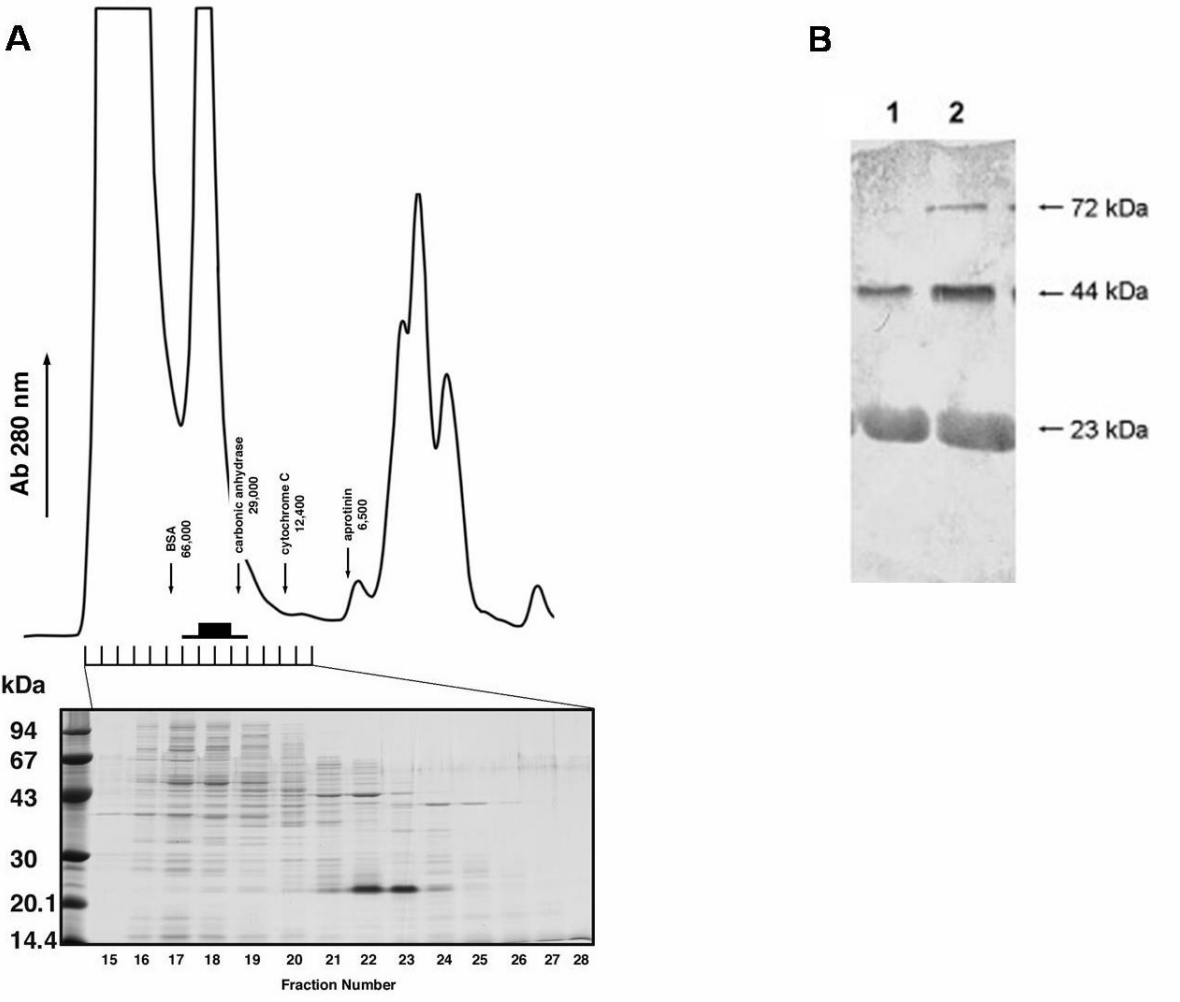
Analysis of self-associating properties of BnDGAT1_(1–116)_His_6 _using gel filtration chromatography and chemical cross-linking. Panel A: Gel filtration chromatography of clarified lysate of bacteria overexpressing BnDGAT1_(1–116)_His_6 _*E. coli *producing BnDGAT1_(1–116)_His_6 _and SDS-PAGE of protein in fractions eluted from the column. IPTG-induced bacteria were resuspended in 30 ml of 100 mM sodium phosphate buffer (pH 7.4) containing 150 mM NaCl. The suspension was passed through a French press three times at 20,000 p.s.i.. The suspension of ruptured bacteria was centrifuged at 13,000 *g *for 15 min to obtain a clarified lysate. The Superdex 75 HR 10/30 column was equilibrated with extraction buffer (100 mM sodium phosphate, pH 7.4 containing 150 mM NaCl) and operated at room temperature with a flow rate of 0.5 mL/min using an FPLC system. One hundred microliters of extract were applied to the column and 1 ml fractions were collected during elution. Ten microliter aliquots were analyzed by SDS-PAGE using a separating gel prepared with a concentration of 15% monomer and 1.1% cross-linker. The column and SDS gel were calibrated with molecular mass markers. Panel B: Chemical cross-linking of BnDGAT1_(1–116)_His_6 _monitored by Western blotting of BnDGAT1_(1–116)_His_6 _before (lane 1) and after (lane 2) treatment with DMS. BnDGAT1_(1–116)_His_6 _(500 μg/mL) was cross-linked in the presence of 4 mg DMS per ml in 0.2 M triethanolamine-HCl at pH 8.5. Cross-linking reactions were allowed to proceed at room temperature for 3 h. The reactions were quenched with 2 × SDS loading buffer and boiled for 5 min prior to application of 10 μL samples to an SDS-PAGE gel, which was prepared using a concentration of 10% total monomer and 1.1% cross-linker.

Analysis of the molecular mass of BnDGAT1_(1–116)_His_6 _under non-denaturing conditions was conducted using gel filtration chromatography of clarified lysate of ruptured bacteria expressing the N-terminal fragment (Figure 4A). Analysis of protein in the fractions eluting from the column by SDS-PAGE indicated that BnDGAT1_(1–116)_His_6 _eluted between the elution positions of carbonic anhydrase (29 kDa) and BSA (66 kDa).

Chemical cross-linking of BnDGAT1_(1–116)_His_6 _was conducted using dimethylsuberimidate (DMS) to further probe the self-associating properties of BnDGAT1_(1–116)_His_6_. Species generated by chemical cross-linking were resolved by SDS-PAGE and visualized by Western blotting using polyclonal antibodies which recognized an epitope in residues 21–35 in the N-terminal region of the polypeptide (Figure 4B). The control lane (lane 1) of the Western blot exhibited immunoreactive proteins with apparent molecular masses of 44 and 23 kDa, respectively. The putative 44 kDa dimer could not be visualized by Coomassie Blue staining suggesting that the immunoreactivity of the dimer was considerably greater than that of the monomer. Treatment of BnDGAT1_(1–116)_His_6 _with chemical cross-linking agent, however, resulted in the clear visualization of an additional band with molecular mass of 72 kDa (lane 2), which may represent tetramer, and an enhancement in the amount of putative 44 kDa dimer. The tetramer was also not visible in Coomassie Blue-stained gels. A very faint immunoreactive band of 72 kDa was also present at this position in lane 1.

### The N-terminal region of BnDGAT1 is localized to the cytosolic side of the ER

**Figure 5:**
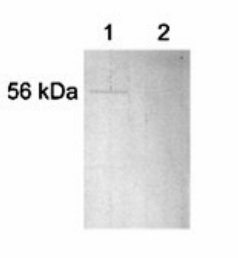
Western blot of microsomal BnDGAT1 before (lane 1) and after Proteinase K treatment (lane 2). Microsomes were prepared from microspore-derived cell suspension cultures of B. napus L. cv Jet Neuf according to Byers et al. [27]. Microsomes containing 1 mg protein were treated for 30 min on ice with 10 μg Proteinase K. The hydrolytic reaction was terminated with 0.1 mg phenylmethylsulfonyl fluoride per mg Proteinase K and incubated on ice for a further 5 min according to Moromoto et al. [51]. Samples were diluted to 1 mL with 10 mM HEPES-NaOH (pH 7.4) and centrifuged for 20 min at 220,000 g at 4°C. The pellet was treated with 150 μl SDS-PAGE sample buffer and boiled for 2 min, and 25 μL were subjected to SDS-PAGE (12% total monomer and 1.1% cross-linker concentration).

Four different computer programs (HMMTOP, TMHMM, TOPPRED and TMPRED) for assessing the topology of membrane-bound proteins indicated that the hydrophilic N-terminal segment of BnDGAT1 resided on the cytosolic face of the ER. An immunochemical approach was used to provide experimental evidence for the location of the hydrophilic N-terminal segment of BnDGAT1 in microsomal vesicles. Polyclonal antibodies raised against a chemically synthesized amino acid sequence representing the peptide segment 21-LDRLHRRKSSSDSSN-35 within BnDGAT1 recognized a 56 kDa polypeptide in SDS-solubilized microsomes (Figure 5, lane 1). This molecular mass was in close agreement with the molecular mass of 56.9 kDa calculated from the deduced amino acid sequence of BnDGAT1 [14]. Pre-treatment of microsomes with Proteinase K, however, resulted in the loss of signal for BnDGAT1 on the Western blot (Figure 5, lane 2) suggesting that the N-terminal segment of the enzyme was digested by the proteinase in microsomal vesicles. This experiment confirmed the cytosolic location of the N-terminal segment of BnDGAT1.

## Discussion

Most of the information about the relationship between structure and function in type 1 DGAT is based on inferences made about proposed functional regions in the enzyme that have been defined by molecular signatures in the sequence data. A detailed 3-dimensional picture of DGAT1 is, however, dependent on the availability of purified enzyme for either crystallization/x-ray diffraction and/or NMR evaluation of structure. Although the type 1 enzyme has been cloned from several sources, the multiple transmembrane segments probably imparted hydrophobicity problems which have impeded purification of the enzyme [1]. In order to gain some insight into structure/function in DGAT1, we generated a poly-His-tagged fragment representing the hydrophilic N-terminal segment of the enzyme from *B. napus*. Attempts to induce the synthesis of the entire poly-His-tagged enzyme in *E. coli *were unsuccessful. The fact that BnDGAT1_(1–116)_His_6 _displayed an anomalously high molecular mass during SDS-PAGE might be related to an unusual conformation for the polypeptide and/or deviation in the degree of SDS-binding by the polypeptide following thermal denaturation in the presence of SDS. Anomalous behavior during SDS-PAGE has been reported for many other proteins [28-31] The ability to crystallize BnDGAT1_(1–116)_His_6 _has set the foundation for future studies of the structure of this fragment. The very fact that a portion of DGAT1 was crystallized represents a major advance in the study of this enzyme.

The binding of acyl-CoA to BnDGAT1_(1–116)_His_6 _is consistent with the presence of an acyl-CoA binding signature (residues 99–116) in the N-terminal region of BnDGAT1 [13]. This signature is also present in other type 1 plant DGATs [10,17] suggesting that this region may have a common role. The sigmoidal response observed for the binding of acyl-CoAs was not likely an artifact of polyHis-tagging because studies with polyHis-tagged acyl-CoA binding proteins from *Arabidopsis *have demonstrated typical hyperbolic saturation in binding studies with radiolabeled acyl-CoAs [32,33]. The hydrophilic N-terminal region of BnDGAT1 would likely interact with the cytosolic acyl-CoA pool because both transmembrane prediction programs and Proteinase K-protection experiments determined that this region was located to the cytosolic side of the ER. The acyl-CoA binding site in the N-terminal region of BnDGAT1 is probably not the active site of the enzyme. BnDGAT2 does not contain the hydrophilic N-terminus present in BnDGAT1 [13,14]. Thus, the absence of the acyl-CoA binding site in BnDGAT2 suggests that amino residues involved in catalysis are common to both BnDGAT1 and BnDGAT2 because cDNAs encoding both of these enzymes were functionally expressed in yeast [13]. The acyl-CoA binding site in the N-terminal segment of BnDGAT1 might represent a regulatory motif on the enzyme or, conversely, a site for trapping cytosolic acyl-CoA for eventual use by the catalytic site. Substantial binding of thioester occurred in the range of 3 – 6 μM acyl-CoA, which is the estimated physiological concentration range of the acyl-CoA pool in developing zygotic embryos of *B. napus *[34]. The binding of acyl-CoA by BnDGAT1_(1–116)_His_6 _occurs over similar concentrations of thioester observed in substrate saturation curves for microsomal DGAT activity from microspore-derived embryos [35] and cell suspension cultures [27] of *B. napus*. As well, the affinity (based on dissociation constant) of BnDGAT1_(1–116)_His_6 _for acyl-CoA is in the same order of magnitude as has been reported for a soluble recombinant acyl-CoA binding protein from developing seeds of *B. napus *[36]. It has been proposed that acyl-CoA binding proteins might modulate acyltransferase activity, perhaps by affecting the delivery of thioester to the enzyme [1].

The competition study involving the introduction of unlabeled CoA or oleoyl-CoA into a mixture containing radiolabeled oleoyl-CoA suggests that the CoA component of the thioester has a major role in determining binding. CoA has previously been shown to enhance DGAT activity in microsomes from microspore-derived cell suspension cultures of *B. napus *[27]. The effect of free CoA in displacing oleoyl-CoA suggests that CoA might act as a negative effector of acyl-CoA action within the N-terminal region of BnDGAT1. Although the thioester interacted with the BnDGAT1 fragment in a cooperative fashion, this study has not linked the binding process to the status of BnDGAT1 activity. Therefore, the relative roles of acyl-CoA and free CoA, potentially regulating BnDGAT1 activity, remain to be elucidated. Furthermore, it is important to note that the effects of CoA on DGAT activity in *B. napus *cultures observed by Byers et al. [27] were based on experiments with microsomes, which may have contained a mixture of type-1 and type-2 DGAT activity, making it difficult to ascribe the stimulatory effect of CoA solely to BnDGAT1 activity. The difference in the binding affinity of oleoyl-CoA versus erucoyl-CoA shown in Figures 2A and 2B indicates that the nature of the fatty acyl moiety is also very important in determining the extent and affinity of binding. Different species of cytosolic acyl-CoA, at various concentrations, might differentially affect the performance of BnDGAT1 in the ER, and this may be further complicated by endogenous CoA concentration. A direct link between the binding events observed for BnDGAT1_(1–116)_His_6 _and catalysis within the functionally active full-length BnDGAT1 will require modification of the putative regulatory site through site-directed mutagenesis.

Collectively, gel filtration chromatography and chemical cross-linking data indicated that BnDGAT1_(1–116)_His_6 _self-associated under non-denaturing conditions. The calculated molecular mass of about 56 kDa for the tetrameric form of BnDGAT1_(1–116)_His_6 _was in agreement with the fragment eluting between 26 and 68 kDa during gel filtration chromatography under non-denaturing conditions. The fact that some putative dimer could be readily detected following SDS-PAGE suggests that a proportion of BnDGAT1_(1–116)_His_6 _retains some native conformation following thermal denaturation in the presence of SDS and reducing agent. Indeed, persistent residual structure in the monomer of human papillomavirus E7 oncoprotein following thermal denaturation in the presence of SDS has been shown to result in anomalous electrophoretic behavior for this protein during SDS-PAGE [31]. The self-association of BnDGAT1_(1–116)_His_6 _is particularly revealing in the light of recent findings which have indicated that both human DGAT1 [21] and mammalian ACAT1 [22] are tetrameric proteins. The N-terminal regions of these mammalian acyltransferases have been shown to play a critical role in the formation of tetramers. Mutations of human DGAT1 devoid of the last 101 amino acid residues from the C-terminus (designated DGAT*sv*) were shown to be catalytically inactive [21]. DGAT*sv*, however, formed dimers and tetramers in chemical cross-linking experiments indicating that the ability to form tetramers resided in the N-terminal region. Mutagenesis studies of an ACAT-related enzyme in yeast, known as Are2p, have provided valuable insights into the structure/function properties of this enzyme [37]. Deletion, truncation and missense mutations implicated a regulatory role for the N-terminal domain of Are2p. The self-associating properties of BnDGAT1 also support the cooperative binding behavior observed in the acyl-CoA binding studies; typically, communication between subunits is required to achieve cooperativity. Thus, the effect of acyl-CoA concentration and species on the self-association of BnDGAT1 would represent an important line of investigation for the future.

## Conclusion

As a first step in deciphering the structure/function relationships in DGAT1, we have examined the properties of a soluble N-terminal fragment of the enzyme from oilseed rape. We have demonstrated experimentally that the recombinant 13.3 kDa N-terminal fragment binds acyl-CoA in a cooperative fashion. In addition, we have shown that this fragment is capable of self-associating to form a putative dimer and tetramer. The ability to crystallize this fragment represents a major advance in the further characterization of this important membrane-bound enzyme.

## Methods

### Materials and services

Restriction enzymes were purchased from Invitrogen Canada Inc. (Mississauga, ON, Canada). PCR buffer and *Taq *polymerase were from Life Technologies (Burlington, ON, Canada). DNA sequencing was conducted at the University Core DNA Services of the University of Calgary (Calgary, AB, Canada). The vector pET26b(+) was from Novagen (Madison, WI, USA) and BL21 (DE3) *E. coli *(Stratagene, La Jolla, CA, USA). Other molecular reagents were of the highest quality and obtained from Fisher Scientific (Nepean, ON, Canada). Ni^2+^-nitrilotriacetic acid (NTA)-agarose was from Qiagen (Mississauga, ON, Canada). MALDI-TOF mass spectrometry for analysis of polypeptide molecular mass was conducted at the Institute for Biomolecular Design at the University of Alberta (Edmonton, AB, Canada). Radiolabeled oleic acid (18:1*cis*^Δ9^; 51 mCi/mmol) and erucic acid (22:1*cis*^Δ13^; 57 mCi/mmol) were from Amersham Canada, Oakville, ON, Canada. Acyl-CoAs were synthesized from radiolabeled fatty acids using acyl-CoA synthetase [38]. Lipidex-1000 was from the Packard Instrument Company (Meriden, CT, U.S.A.). Ecolite™ (+) biodegradable scintillant was from ICN Biomedicals (Irvine, CA, U.S.A.). The Superdex 75 HR 10/30 column was from GE Healthcare, (Baie D'Urfe, QC, Canada). Proteinase K was obtained from Invitrogen Canada Inc. All other biochemicals were purchased from Sigma-Aldrich Canada (Oakville, ON, Canada).

### Cloning of the cDNA fragment encoding BnDGAT1_(1–116)_His_6_

Most molecular genetic procedures used were adapted from Ausubel et al. [39]. Analysis of the deduced amino acid sequence of BnDGAT1 indicated that residues 1–116 formed a relatively hydrophilic N-terminus [13,14]. The plasmid pGEM containing *BnDGAT1 *cDNA (GenBank accession no. AF164434) was amplified, via PCR, in the region encoding amino acid residues 1–116 using the forward and reverse primers 5'-TCATATGGCGATTTTGGATTCTGGAG-3' and 5'-GCGGCCGCATGGCTTTGTTTGAAGAT-3', respectively. The blunt ended PCR product was purified by agarose gel electrophoresis [40] and ligated into a pUC vector that had been linearized with *Sma*I. The ligated product was used to transform competent DH5α *Escherichia coli*, which were subsequently grown on β-D-thiogalactosidel/LB_amp _plates. Putative transformed colonies were transferred to patch plates and after further growth, the resultant colonies were tested for the presence of the insert via restriction analysis with *Nde*I and *Not*I. A colony that tested positive for the insert via restriction analysis was sequenced to confirm the construct. The colony was grown overnight in LB_amp _broth, and plasmid DNA was isolated and digested with *Nde*I and *Not*I. The products of the restriction reaction were run on an agarose gel and the band corresponding to the insert was purified from the agarose gel. The expression vector pET26b(+) was also digested and cleaned in the same manner. The insert and treated vector were ligated to each other and used to transform competent BL21 (DE3) *E. coli*. Selection was performed using LB_kan _plates. A patch plate was generated and colony PCR was performed to test for the presence of the insert. A positive colony was isolated and propagated overnight in LB_kan _broth, and the plasmid DNA was isolated and sequenced.

### Expression and purification of BnDGAT1_(1–116)_His_6_

BL21 (DE3) cells capable of synthesizing BnDGAT1_(1–116)_His_6 _were grown as streak plates for 16 h at 37°C followed by inoculation into 5 mL of LB_kan_. The starter liquid culture was allowed to grow for 3 h at 37°C. Five hundred milliliters of LB_kan _were inoculated with the entire starter culture and the resulting mixture was incubated with vigorous shaking for 5 h at 37°C. The culture was induced with 2 mM (final concentration) IPTG and then incubated overnight at room temperature with shaking. The cells were harvested and centrifuged at 22,100 *g *for 10 min. After this step, all subsequent operations were conducted at 0–4°C. The supernatant was discarded, and the sediment of cells was resuspended in 10 mL of 10 mM HEPES-NaOH buffer (pH 8.0) containing 1 mM 2-mercaptoethanol and 100 mM NaCl. The suspension was passed through a French pressure cell three times at 20,000 p.s.i.. The suspension of ruptured bacteria was centrifuged at 107,000 *g *for 1 h to obtain the soluble subcellular fraction. The supernatant was combined with 1 mL of sedimented Ni^2+^-NTA-agarose, which was pre-equilibrated with 10 mM HEPES-NaOH (pH 8.0). The resulting suspension was incubated with constant shaking, for 30 min. The suspension was then applied to a 0.5 cm diameter polyethylene column with a porous disc at the bottom. After the immobilized nickel ion affinity gel sedimented, the column was washed with equilibration buffer and then eluted sequentially with increasing concentrations of imidazole in equilibration buffer. The column was stripped with 400 mM imidazole in equilibration buffer. Protein concentrations were determined via the Bradford [41] method using BioRad's microassay with BSA as a standard. Eluates were analyzed by SDS-PAGE [42]. The separating gel was prepared using 15% monomer and 1.1% cross-linker concentration, and electrophoresis was conducted for 1.5 h at 100 V. The gel was stained with Coomassie Brilliant Blue R-250. Purified BnDGAT1_(1–116)_His_6_, eluted with 40 mM imidazole, was dialyzed overnight 4°C in 10 mM potassium phosphate buffer, pH 7.4 using #1 Spectra/Por Molecular porous membrane with 6,000–8,000 mol. wt. cut-off (Rancho Dominguez, CA, USA). The dialyzed BnDGAT1_(1–116)_His_6 _solution (~1 mg/mL) was frozen with liquid nitrogen in small aliquots in microcentrifuge tubes and stored at -80°C.

### DLS of BnDGAT1_(1–116)_His_6_

DLS was conducted using a Protein Solutions Light Scattering Instrument (DynaPro International Ltd., Crownhill, Milton Keynes, England, UK). The enzyme fragment was concentrated to about 5 mg/mL using a centrifuge-driven ultrafiltration cell equipped with 3000 mol. wt. cut-off membrane (Filtron Technology Corporation, Clinton, MA, USA). The concentrated protein solution was dialyzed overnight against 10 mM HEPES-NaOH, pH 7.4. Immediately prior to DLS, 60 μL of the protein solution was centrifuged at 12,000 *g *for 5 min followed by filtration through a 0.1 μm membrane. DLS was conducted at 20°C using a 12 μL cell.

### Crystallization of BnDGAT1_(1–116)_His_6_

Purified BnDGAT1_(1–116)_His_6 _was concentrated to 12 mg/mL by ultrafiltration and dialyzed overnight into various buffered environments. Initial screening for crystallization conditions was conducted using vapor diffusion and hanging drops.

### Lipidex-1000 binding assays

Binding of radiolabeled acyl-CoA to BnDGAT1_(1–116)_His_6 _was conducted using a Lipidex-1000 binding assay [43]. Binding assays were conducted in duplicate in 10 mM potassium phosphate buffer, pH 7.4, at 30°C with [1-^14^C]acyl-CoAs (oleoyl- and erucoyl-CoA) and 0.2 μM protein. Incubations were set up without protein to correct for acyl-CoA that was not adsorbed to the Lipidex-1000.

### Analysis of acyl-CoA-BnDGAT1_(1–116)_His_6 _interaction by gel electrophoresis

Binding of radiolabeled erucoyl-CoA to BnDGAT1_(1–116)_His_6 _was also analyzed using gel electrophoresis in combination with phosphorimaging based on an approach described by Engeseth et al. [44]. Five microliters of BnDGAT1_(1–116)_His_6 _(7.5 μg) were combined with 3.3 μL of 180 μM [1-^14^C]erucoyl-CoA (57 mCi/mMol) in a final volume of 15 μL in a buffer environment of 10 mM potassium phosphate (pH 7.4). This incubation was set up in duplicate and included a control incubation prepared without protein. The mixture was incubated for 30 min at room temperature and then subjected to electrophoresis on a 12% separating gel (1.1% cross-linker) using Laemmli buffers [42] without SDS and without a spacer gel. Prior to sample application, 10 μL of a modified loading buffer were added to the 15 μL incubation mixture and the entire sample was subjected to gel electrophoresis. The modified loading buffer consisted of 150 μL glycerol, 40 μL of 0.5% bromophenol blue and 660 μL of 10 mM potassium phosphate buffer. Samples were not boiled prior to application to the gel. Electrophoresis was conducted at 100 V (constant voltage) until the dye front reached the bottom of the gel. One gel section containing lanes for the control and one interaction test was subjected to phosphorimaging using a Cyclone Storage Phosphor System (PerkinElmer Life and Analytical Sciences, Woodbridge, ON, Canada). The remaining gel segment was stained with Coomassie Blue R-250. The resulting phosphorimage was aligned with the gel stained for protein.

### Gel filtration chromatography of BnDGAT1_(1–116)_His_6_

Superdex 75 HR 10/30 gel filtration chromatography was used to evaluate the native molecular mass of BnDGAT1_(1–116)_His_6 _in an extract prepared from *E. coli *overexpressing the N-terminal fragment. In this case, the *E. coli *producing BnDGAT1_(1–116)_His_6 _were resuspended in 30 mL of 100 mM sodium phosphate buffer (pH 7.4) containing 150 mM NaCl. The suspension was passed three times through a French pressure cell at 20,000 p.s.i.. The suspension of ruptured bacteria was centrifuged at 13,000 *g *for 15 min to obtain a clarified lysate. The column was eluted at room temperature with extraction buffer at a flow rate of 0.5 ml/min using a Fast Protein Liquid Chromatography (FPLC) system (GE Healthcare Canada Inc.). One hundred microliters of extract were applied to the column. One milliliter fractions were collected and 10 μL aliquots were analyzed by SDS-PAGE. The column and SDS gel were calibrated with molecular mass markers.

### Cross-linking of BnDGAT1_(1–116)_His_6_

BnDGAT1_(1–116)_His_6 _was dialyzed into 0.2 M triethanolamine-HCl, pH 8.5. Cross-linking [45] was conducted at a protein concentration of 500 μg/mL using 4 mg DMS (dihydrochloride)/mL in 0.2 M triethanolamine-HCl buffer (pH 8.5) for 3 h at room temperature. DMS was dissolved in buffer immediately before use. Cross-linking reactions were quenched with 2 × SDS loading buffer and boiled for 5 min prior to SDS-PAGE. Western blotting was conducted according to Nykiforuk et al. [13] using polyclonal antibodies raised against a peptide of 15 of amino acid residues (21-LDRLHRRKSSSDSSN-35) representing a segment within the N-terminal region of BnDGAT1.

### Predicting membrane topology for BnDGAT1

Four programs from the Expasy Molecular Biology Server [46] were used for topology predictions of the deduced BnDGAT1 amino acid sequence: HMMTOP [47], TMHMM [48], TOPPRED [49] and TMPRED [50].

### Preparation of microsomes from cell suspension cultures of *B. napus*

Microspore-derived cell suspension cultures of *B. napus *L. cv Jet Neuf were maintained according to Orr et al. [51]. Microsomes sedimenting at 10,000–100,000 *g *were prepared from 1 g of frozen cultured cells according to Byers et al. [27]. Microsomes were resuspended in 10 mM HEPES-NaOH buffer (pH 7.4) and homogenized with a Potter Elvehjem homogenizer, and then centrifuged for 20 min at 220,000 *g *at 4°C. The pellet was resuspended in 1 mL HEPES-NaOH buffer (pH 7.4) and the protein content was determined.

### Proteinase K treatment of microsomes and western blotting for BnDGAT1

Microsomes containing 1 mg protein were treated with 0.01 mg Proteinase K for 30 min on ice according to Morimoto et al. [52]. The reaction was terminated by adding 0.1 mg phenylmethylsulfonyl fluoride per mg Proteinase K followed by 5 min of additional incubation on ice. Samples were diluted to 1 ml with 10 mM HEPES-NaOH buffer (pH 7.4) and centrifuged at 220,000 *g *for 20 min at 4°C. The resulting pellets were resuspended in 150 μl SDS loading buffer, boiled for 5 min and 25 μL were subjected to SDS-PAGE (12% total monomer and 1.1% cross-linker concentration). Western blotting was performed according to Nykiforuk et al. [13].

## Abbreviations

ACAT – acyl-CoA:cholesterol acyltransferase

BnDGAT1 – *Brassica napus *diacylglycerol acyltransferase-1

BnDGAT1_(1–116)_His_6 _– poly-His tagged recombinant hydrophilic N-terminal fragment of *Brassica napus *diacylglycerol acyltransferase-1

DAG – *sn*-1,2-diacylglycerol

DGAT – acyl-CoA:diacylglycerol acyltransferase

DLS – dynamic light scattering

DMS – dimethyl suberimidate

FPLC – Fast Protein Liquid Chromatography

IPTG – isopropyl β-D-thiogalactoside

NTA – nitrilotriacetic acid

TAG – triacylglycerol

## Authors' contributions

RJW designed the overall study, conducted initial experiments on the cloning and expression of BnDGAT1_(1–116)_His_6_, performed Lipidex-1000 binding assays and prepared most of the initial draft of the manuscript. MM was in involved in the preparation of BnDGAT1_(1–116)_His_6_, conducted binding assays and contributed to some of the writing. SJS provided guidance on protein expression in bacteria and assisted with presentation of the data. NAP, WBW, CLN, MMM, and AL provided valuable expertise in molecular genetic methods and input into the development of the manuscript. TLB assisted in the design of cross-linking studies and also performed these studies. PSB was involved in the cloning and expression of BnDGAT1_(1–116)_His_6_. SCM developed conditions for crystallizing BnDGAT1_(1–116)_His_6_. NAF conducted computer-based analysis of the topology of BnDGAT1 and conducted immunochemical studies on the localization of the N-terminus. BJT and TLF-S were involved in the design and execution of gel electrophoresis experiments to assess the acyl-CoA binding capability of BnDGAT1_(1–116)_His_6_.
